# SciKit-Surgery: compact libraries for surgical navigation

**DOI:** 10.1007/s11548-020-02180-5

**Published:** 2020-05-20

**Authors:** Stephen Thompson, Thomas Dowrick, Mian Ahmad, Goufang Xiao, Bongjin Koo, Ester Bonmati, Kim Kahl, Matthew J. Clarkson

**Affiliations:** Wellcome/EPSRC Centre for Interventional and Surgical Sciences, UCL, London, UK

**Keywords:** Image-guided surgery, Platform, Software, Python, Surgical navigation

## Abstract

**Purpose:**

This paper introduces the SciKit-Surgery libraries, designed to enable rapid development of clinical applications for image-guided interventions. SciKit-Surgery implements a family of compact, orthogonal, libraries accompanied by robust testing, documentation, and quality control. SciKit-Surgery libraries can be rapidly assembled into testable clinical applications and subsequently translated to production software without the need for software reimplementation. The aim is to support translation from single surgeon trials to multicentre trials in under 2 years.

**Methods:**

At the time of publication, there were 13 SciKit-Surgery libraries provide functionality for visualisation and augmented reality in surgery, together with hardware interfaces for video, tracking, and ultrasound sources. The libraries are stand-alone, open source, and provide Python interfaces. This design approach enables fast development of robust applications and subsequent translation. The paper compares the libraries with existing platforms and uses two example applications to show how SciKit-Surgery libraries can be used in practice.

**Results:**

Using the number of lines of code and the occurrence of cross-dependencies as proxy measurements of code complexity, two example applications using SciKit-Surgery libraries are analysed. The SciKit-Surgery libraries demonstrate ability to support rapid development of testable clinical applications. By maintaining stricter orthogonality between libraries, the number, and complexity of dependencies can be reduced. The SciKit-Surgery libraries also demonstrate the potential to support wider dissemination of novel research.

**Conclusion:**

The SciKit-Surgery libraries utilise the modularity of the Python language and the standard data types of the NumPy package to provide an easy-to-use, well-tested, and extensible set of tools for the development of applications for image-guided interventions. The example application built on SciKit-Surgery has a simpler dependency structure than the same application built using a monolithic platform, making ongoing clinical translation more feasible.

## Introduction

The development of novel algorithms for image-guided interventions (IGI) brings together research in six areas: medical imaging, medical image computing, registration, tracking, visualisation, and user interface design. Researchers aiming to build and test clinical applications incorporating novel algorithms can benefit significantly by using software platforms or toolkits that provide ready-made functionality in each of these six areas and the connections between them [23]. There are open-source and commercial platforms that meet these needs for the purpose of rapid prototyping and development of clinical applications. However, we have found that the use of such platforms can hinder translation to approved clinical software, due to the software engineering resources required to turn a prototype application into clinically useable software.

Applications built on general platforms will only use a tiny fraction of the platform’s functionality. In addition to the code implementing the unused functionality, this comes at the cost of additional dependencies. Therefore, using a platform for developing a clinical application introduces a lot of code and dependencies that are not necessary for the application. The amount of resources required to certify and maintain a clinical application scales with both the complexity of the application and the number and size of dependencies. SciKit-Surgery aims to avoid unnecessary code and dependencies by providing tools for surgical innovation that are more independent.

The SciKit-Surgery libraries have been developed to support the accelerated translation of clinical innovations to deployable clinical applications. We phrase this as going from “bench to bedside” in under 2 years [19]. SciKit-Surgery libraries help enable this by reducing code complexity and the number of dependencies, whilst providing enough features to support rapid development of testable clinical applications. In contrast to other work in this area, SciKit-Surgery

- is a set of stand-alone libraries not a platform,
- allows researchers to combine individual libraries to create clinical applications without compilation or dependency problems and
- uses Python for most functions.

During early development, the libraries used the working title SNAPPY; however, this was changed to make them easier to find using typical search techniques. The SciKit prefix indicates that they are part of the Python scientific ecosystem, whilst Surgery indicates their intended purpose.

The rest of this paper is structured as follows. Section 2 describes existing platforms and toolkits used for IGI applications and compares them with SciKit-Surgery. Section 3 discusses the design choices made when developing SciKit-Surgery. Section 4 introduces SciKit-Surgery’s current component libraries. Section 5 illustrates some of the benefits of SciKit-Surgery using two example applications.

## Background

Wolf [25] and Cleary [6] provide overviews of the existing software for image-guided interventions. The Medical Imaging Interaction Toolkit (MITK) [9] and 3DSlicer [20] are the two most widely used open-source platforms for development of IGI systems. The Image-Guided Surgery Toolkik (IGSTK) [5] implemented many tools for image-guided interventions and could be integrated with MITK [16] and 3DSlicer; however, IGSTK is no longer under development. The Public Software Library for UltraSound imaging research (PLUS) [15] implements numerous hardware interfaces and messaging. PLUS can be integrated with SlicerIGT[23] to provide IGI applications. Other platforms include CamiTK [8], NifTK [3], IBIS [7], CISST [4], and CustusX [1].

Table 1 lists these IGI platforms and compares them based on the size of the source code. To measure the size of the platforms, we used cloc,^1^ to count the lines of source code in each project. We counted lines of code on the master branch of each platform, as of October 2019. Lines of source code does not give a particularly robust comparison between projects; however, it is sufficient to give an indication of the overall complexity of the platforms.

An alternative approach to the development of IGI applications is to use a more general-purpose scientific package like MATLAB[17], which provides an easy-to-use set of user interface tools together with inbuilt and third-party libraries for interfacing with various devices. For example, Hu et al. [13] demonstrated an application for targeted prostate biopsy using Matlab for the user interface, whilst Medviso [12] provided commercial medical imaging analysis software built on MATLAB. However, we have excluded MATLAB from Table 1 as it is not open source.

**Table 1:** Comparable sizes of other platforms, in kilo lines of code(kLoC), measured using cloc version 1.76

Library	Size (kLoC)
3DSlicer	C$++$: 298.77 Python : 33.34
SlicerIGT	C$++$: 13.73 Python : 2.78
PlusLib	C$++$: 92.89 C : 11.05
NifTK	C$++$: 206.57
MITK	C$++$: 419.17
CustusX	C$++$: 113.61
IBIS	C$++$: 48.50
CISST	C$++$: 135.68 Python : 3538
CamiTK	C$++$: 63.69

Platforms like 3DSlicer, MITK, and MATLAB have been successfully used to develop prototype clinical applications. Most platforms provide the capacity to rapidly prototype clinical applications, using either scripting interfaces or modular plugin-based extension. We have previously used MITK’s modular architecture to develop the NifTK [3] platform. NifTK was then used to create an augmented reality application for laparoscopic liver surgery [22]. However, it remains uncommon for prototype clinical applications to progress beyond research papers and into clinical products. The use of the above platforms can create difficulty during translation to approved medical software, due in part to the difficulty in identifying essential components and dependencies.

**Table 2:** The SciKit-Surgery libraries were developed with three aims in mind

SciKit-Surgery Design Aims	
1	Enable novel algorithms to be built into applications that can be rapidly deployed to theatre
2	Minimise reimplementation during translation from proof of concept to multicentre trials
3	Enable dissemination of high-quality implementations of research algorithms

**Table 3:** The aims were translated into a set of design guidelines that can be followed when developing an individual SciKit-Surgery library

SciKit-Surgery Design Guide	
1	Development driven by users’ needs
2	Do one thing and do it well
3	Functions and classes with Python interfaces using NumPy data structures
4	Installable directly from PyPI, using pip
5	Libraries fully documented and examples provided
6	Packages are small with clear instructions on how to contribute to development
7	Packages are independent, with minimal dependencies, and maximum orthogonality
8	Use version control, issue tracking, and continuous integration testing
9	Template for library development and encourage wider adoption

Although it is not entirely reasonable to compare the size of the SciKit-Surgery libraries with some of the other platforms used for IGI, it is helpful for putting them into context. The libraries listed in Table 1 implement a much larger tool set, which comes at the cost of much greater complexity. In Sect. 4, we demonstrate that the SciKit-Surgery libraries are much smaller and have fewer direct dependencies than the platforms listed in Table 1.

It is also notable that all the platforms listed in Table 1 are written primarily in C$++$. We have found it increasingly hard to recruit researchers with the skills or willingness to develop in C$++$, making development and translation based on these platforms more difficult.

Although the platforms listed in Table 1 require a degree of effort in getting familiar with (and in most cases compiling) the platform, once this is done they offer convenience and completeness for the rapid prototyping of medical applications. However, we argue that their size, internal complexity, and choice of language makes translation to an approved clinical product difficult.

## SciKit-Surgery design aims and guidelines

SciKit-Surgery originated within the Wellcome EPSRC Centre for Interventional and Surgical Sciences (WEISS), to support clinical translation and innovation. We set three aims for the SciKit-Surgery libraries and used these aims to inform the SciKit-Surgery design guide. Tables 2 and 3 enumerates the aims and design guide. In this section, we discuss how the aims were turned into design guidelines.

### Goal 1: rapid deployment to theatre

SciKit-Surgery is aimed at researchers developing novel algorithms to perform a specific task within a clinical environment. Typically, the algorithm can be implemented in a few hundred lines of code; however, there is a need for supporting infrastructure to supply data input and output, visualisation, and hardware and user interfaces. As discussed in Sect. 2, researchers already have a wide choice of platforms that can fulfil this requirement. In our experience, the main drivers for the adoption of a particular platform are:

1. Implementation of the required functionality.
2. Familiarity with the programming language used.
3. Ease of installation on the researcher’s system.
4. Number of users amongst the researcher’s peers.

Most existing platforms meet the first driver, usually providing more functionality than will be necessary for a given application. Additional functionality introduces additional code complexity and dependencies. Therefore, we set the first SciKit-Surgery design guideline to be “Needs driven development”. Rather than setting out to deliver a platform that meets all users’ as yet unknown needs, new functions and classes are only implemented when they are needed for a particular project.

To avoid the individual libraries and their dependencies steadily growing as new needs are identified, the SciKit-Surgery libraries follow the Unix philosophy of doing one thing and doing it well [18]. When a new feature is requested, careful though is given to whether it should fit within an existing library or a new SciKit-Surgery library should be implemented.

There are a variety of approaches towards meeting the second driver. Past attempts to get researchers to develop in C$++$ have failed as most have not had exposure to C$++$ at undergraduate level. MATLAB has a historic advantage as many researchers in the field have had exposure to it at undergraduate level. However, we have found that Python is now widely used in research and education, so we made the decision to adopt a Python interface, design guideline 3. This does not preclude libraries written in other languages; however, they should provide a Python interface, so that they can be easily integrated into applications.

We have found that the third driver is the one that many platforms struggle with. Whilst in some cases pre-built binary applications are available, once the user seeks to develop their own applications it is often necessary to compile the platform from source. We have found that this can be off putting for potential users. A method to compile robustly across multiple operating systems and compilers remains elusive. Most user’s first experience of compilation results in failure. Whether or not they persist depends on the strength of the other use drivers. For this reason, we decided to avoid libraries requiring user compilation. The Python packaging index^2^ provides a well-integrated platform to handle dependencies and install both pure Python and binary executable software libraries, leading to design guideline 4. To make using the libraries intuitive to use, we also ensure they are fully documented and conform to PEP8 standards using static code analysis.^3^ In addition to examples contained with each library (guideline 5), there are two tutorials to date (see Sect. 7) to help dissemination.

We have actively tried to keep each library as compact and atomic as possible. When confronted with a large platform [3], many users are deterred from contributing due to the difficulty of working out where their algorithm belongs and a fear of breaking something elsewhere in the platform. By keeping the constituent libraries small, with a clear purpose and consistent structure (design guideline 6), we have seen a greater willingness of users to contribute. A good example of a researcher-led contribution is the scikit-surgeryspeech library, developed as a stand-alone project during a summer internship.

### Goal 2: reduce the need for reimplementation

Applications such as LumpNav [23] and SmartLiver [22] are representative of typical clinical research prototypes. These applications consist of between 500 and 4000 lines of code, written by a single researcher or small ($<4$) team, utilising pre-existing platforms. The resulting applications are sufficient to support single user clinical trials and publication in technical and clinical journals. However, to prove safety and efficacy it is necessary to progress to multicentre clinical trials, ultimately aiming for certification for medical use. This next stage of development requires a level of quality control and testing that is often fatal to the application, leading to the “Valley of Death” [2].

Turning a prototype built on top of a platform into a stable, testable, application is a difficult problem. To make the task manageable, it is useful to be able to strip away unused functionality from the platform. Disassembling a monolithic platform is substantially harder than building an application from many components [14]. The alternative approach is to reimplement the application from the ground up, which is also very resource intensive.

SciKit-Surgery has been designed to reduce the need for re-engineering at this stage of translation. By keeping the libraries compact and maintaining orthogonality between them (design guideline 7), it becomes possible to assemble them into a minimal functional application that forms the skeleton for ongoing development. This fits the “Tracer Bullet” approach to development described by Thomas and Hunt [14].

SciKit-Surgery libraries are developed in line with the U.S. Food and Drug Administration’s guidelines on software development for medical applications [24]. SciKit-Surgery design guideline 8 specifies version control, issue tracking, and continuous integration testing via GitLab, to make integration into a clinical product as efficient as possible.

### Goal 3: dissemination of research

The third aim has three motivations:

- maximise the impact of research, regardless of any clinical translation,
- improve the software development skills of researchers and students,
- and to encourage researchers to contribute to SciKit-Surgery.

Alongside SciKit-Surgery, we have developed the Python and C$++$ templates, design guideline 9. These are the starting point for most SciKit-Surgery libraries, saving time and maintaining a familial resemblance across libraries. The templates are also designed to be used by researchers as a base to implement their algorithms. The templates support code quality via static code analysis and unit testing and simplify publishing to pypi.org. By using the templates, researchers ensure that their algorithms can be incorporated into clinical applications using the SciKit-Surgery libraries. Furthermore, the researchers’ code becomes a stand-alone library that can be readily shared and used by other researchers.

A good example of the use of the Python template to aid dissemination is provided by Fu et al. [10]. The software used in their paper is now published on PyPI^4^ allowing other researchers to replicate the results.

By encouraging researchers to become contributors, we hope to make SciKit-Surgery sustainable. A key challenge with any software platform is maintenance. Keeping a monolithic platform with multiple dependencies up to date will consume an increasing amount of resources, resulting in the eventual death of the platform. By separating the libraries and encouraging contribution from diverse researchers, we hope to make ongoing maintenance achievable.

## The SciKit-Surgery libraries and architecture

In this section, we introduce the libraries that currently constitute SciKit-Surgery. As discussed in Sect. 3.1, we are operating a needs-based development policy, so the constituent libraries are sufficient to meet our current needs, but will be expanded for future applications.

The most notable absence from a toolkit aimed at medical applications is a DICOM reader or a viewer for voxel images. These functions are not usually necessary for augmented reality applications, where simplifying the display using volumetric segmentations from the original images is useful. Segmentation can be performed with a separate application or even outsourced to external suppliers (e.g. VisiblePatient^5^). In any case, the modular nature of SciKit-Surgery allows existing Python packages for reading and writing DICOM data to be easily used (e.g. https://pypi.org/project/pydicom).

The lack of medical image computing tools marks an important difference to most of the platforms discussed in Sect. 2. Most platforms are medical-imaging toolkits with extensions to support computer-aided interventions, whereas SciKit-Surgery is composed of tools aimed at computer-aided interventions. SciKit-Surgery libraries for medical image computing may be implemented in the future.

Table 4 lists 13 libraries that currently constitute SciKit-Surgery. Within the core libraries, there are at most six primary dependencies, in line with design guideline 7. This can be contrasted with NifTK for example which currently has approximately 35 primary dependencies. We have adopted the naming convention of using the prefix “SciKit-Surgery” followed by a short descriptive title, which fits in well with the existing Python package ecosystem.

**Table 4:** The libraries that currently make up SciKit-Surgery. The two templates are not prefixed with SciKit-Surgery, as they have no surgical functionality. All libraries can be found on pypi.org

Library	Components	Dependencies	Size (kLoC)
*SciKit-Surgery Core Libraries (scikit-prefix omitted for brevity)*			
surgerycore	Common data types, abstract base classes, configuration manager, transform manager, common algorithms	NumPy	0.78
Surgeryimage	Video input, distortion correction, rectification, common image algorithms	NumPy, OpenCV, surgerycore	3.36
Surgeryvtk	Visualisation, surface model loaders, camera models, polydata utilities, widgets	NumPy, VTK, OpenCV, QT(PySide2), surgerycore, surgeryimage	3.98
*SciKit-Surgery Hardware Interface Libraries (scikit-prefix omitted for brevity)*			
Surgery-nditracker	NDI trackers (Polaris, Vega, Aurora)	NumPy, ndicapi, surgerycore	1.62
Surgery-arucotracker	Tracking using ArUco Tags	NumPy, OpenCV, surgerycore	1.55
Surgerybk	BK Medical Ultrasound	NumPy, OpenCV, surgerycore	1.55
*SciKit-Surgery Application Libraries (scikit-prefix omitted for brevity)*			
Surgeryutils	Utility applications, basic overlay window, reslice window, video lag measurement	NumPy, OpenCV, QT(PySide2), surgeryimage, surgeryvtk	1.88
Surgery-tracker-visualisation	Visualisation for surgerytrackers	NumPy, VTK, PySide2, surgeryvtk, surgeryimage, surgerycore, surgeryutils, surgerynditracker, surgeryarucotracker	1.74
Surgery-davinci	Augmented Reality for the DaVinci Robot	NumPy, surgeryvtk, surgeryimage, surgerycore, surgeryutils	1.54
*SciKit-Surgery User Interface Libraries (scikit-prefix omitted for brevity)*			
Surgeryspeech	Speech recognition user interface	pyaudio SpeechRecognition, google-api-python-client, oauth2client, PySide2	1.37
SciKit-Surgery C$++$ Libraries (scikit-prefix omitted for brevity)			
Surgerygpucpp	GPU accelerated image processing	ArrayFire, Boost, Eigen, FLANN, glog, OpenCV, PCL, VTK	2.33
*SciKit-Surgery Library Templates*			
PythonTemplate	Template for native Python SciKit-Surgery Libraries	cookiecutter	1.27
C$++$ Template	Template for C$++$ SciKit-Surgery Libraries	cookiecutter	1.27

All 13 libraries are open source and developed in line with design guideline 8. Continuous integration test status, coverage statistics, and documentation are all linked from the individual library’s PyPI page. Users can submit issues via the libraries’ GitLab or GitHub pages and contribute changes via the usual processes of forking and merge requests. Testing on multiple platforms and build environments is currently handled via tox and GitLab.

Three libraries lie at the core of SciKit-Surgery. scikit-surgerycore is intended to help bind the various libraries together by defining common data types and interfaces. For example, scikit-surgerycore defines an abstract base class for surgery trackers, so in theory applications using scikit-surgerytrackers can easily switch between tracking hardware. scikit-surgeryimage handles the acquisition, calibration, and basic processing of the video images which create the back drop for augmented reality. Due to the necessary processing power required for some algorithms (e.g. surface reconstruction, depth estimation), these will be delegated to specific libraries utilising hardware acceleration, i.e. scikit-surgerygpucpp. scikit-surgeryvtk handles the second part of augmented reality, the positioning and rendering of surface models. scikit-surgeryvtk provides surface models loaders, camera models, and overlay widgets.

As the libraries are under continuous development, we refer the reader to the individual libraries’ documentation (accessible from PyPI) for up to date descriptions of each library’s contents. From PyPI The user is able to quickly see the status of the library, which should show green for testing, coverage, and documentation buttons. Indexes of the functions and classes that a module implements are also available. Static code analysis (pylint) is used to ensure that documentation is kept up to date.

## From SciKit-Surgery libraries to application; two case studies

This section shows, using two examples, how SciKit-Surgery has been used to produce applications used in surgery, enabled the rapid prototyping of novel algorithms, and enabled dissemination. The first of these applications, the SmartLiver surgical guidance application [22], compares the implementation using SciKit-Surgery, with a previous implementation as a plugin within the NifTK [3] platform. The point of the comparison is to illustrate that although the size of the actual applications is largely independent of the underlying libraries used, the use of the SciKit-Surgery libraries reduces the size and complexity of dependencies. The second application, SnappySonic [21], was developed to support public engagement and education and is included to illustrate how SciKit-Surgery supports rapid development and dissemination.

### SmartLiver augmented reality liver surgery

Previous work in our laboratory [22] on an augmented reality guidance system for liver surgery has yielded promising clinical results and continued funding to develop the system into a commercially viable guidance system. Here, we discuss the reimplementation of the SmartLiver application using the SciKit-Surgery libraries, to demonstrate by example how the SciKit-Surgery libraries help meet the second development goal from Tables 2 and 3.

**Fig. 1:**
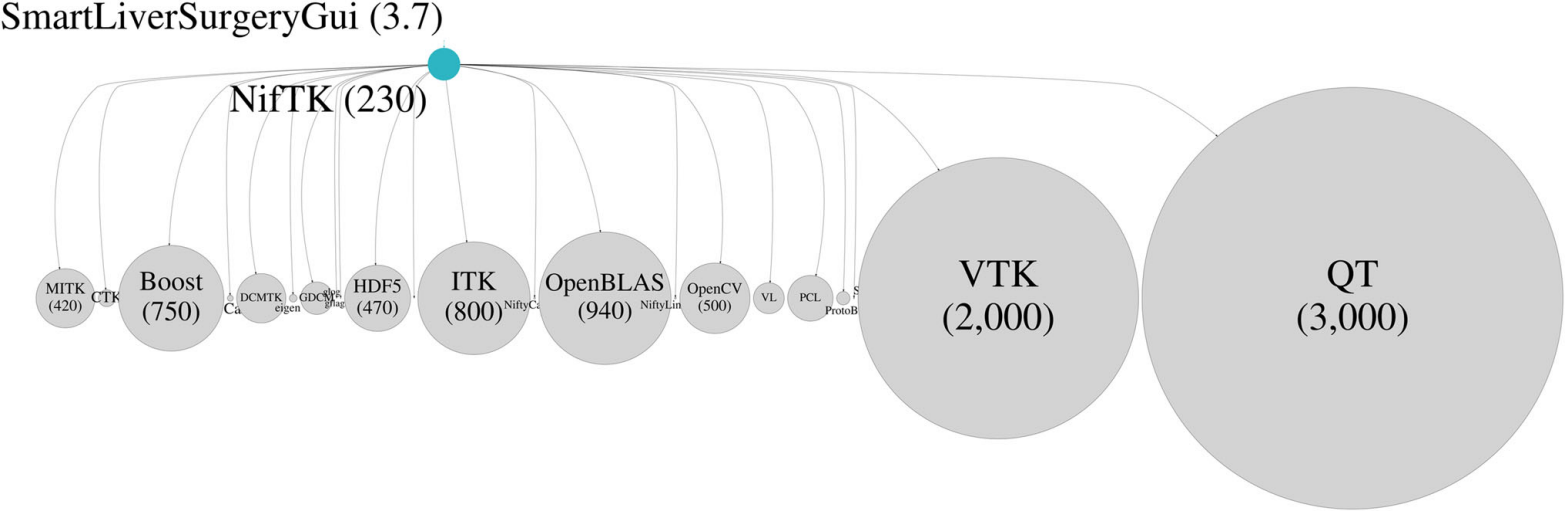
Use of a platform such as NifTK allowed the SmartLiver augmented reality guidance system to be written in approximately 3.7 k lines of code, with a single dependency on the NifTK platform, 230 k lines of code. In common with other platforms (and the SciKit-Surgery libraries), NifTK is built on top of further dependencies, shown scaled by size along the bottom of the figure. The direct dependencies of NifTK total 10.36M lines of code

Our previous implementation of the software took the form of a plugin to the NifTK software platform [3]. This approach enabled a clinical application to be developed and tested in vivo within the time frame of a research grant (3 years). The plugin itself consists of approximately 3.7 k lines of codes, while NifTK has around 230 k lines of code with dependencies on 10.36 M lines of code, see Fig. 1. These numbers are remarkably similar to those for other applications in the literature [23].

**Fig. 2:**
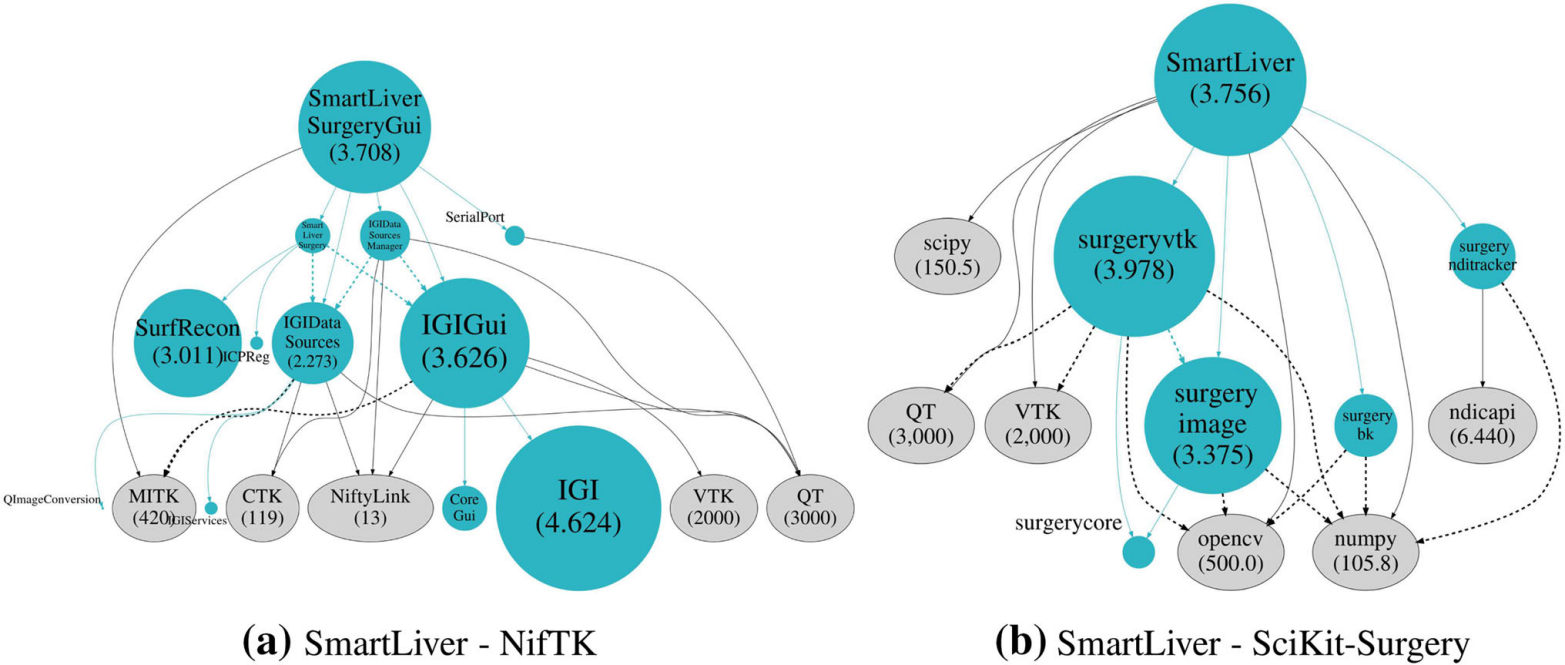
First- and second-order modular dependencies for SmartLiver implemented using NifTK **a** and SciKit-Surgery **b**. Dependencies that form part of NifTK or SciKit-Surgery are shown in light blue and are scaled by size. Third-party dependencies are shown in grey and are not scaled by size, as they (principally VTK and QT) would dwarf the other dependencies. Cross-dependencies (dependencies between first-order dependencies) are highlighted using dashed lines. Where space permits the library size in thousands of lines of code is shown in brackets

If the SmartLiver system were ready to be deployed, it could be turned into a clinical product by freezing all dependencies and supplying the application on a clinical grade computer for use in theatre. However, our research in this area is ongoing, requiring a live platform. NifTK, in common with other IGI platforms, has a small developer and user base, relative to its size, and is dependent on external grant funding. Development of NifTK has stalled due to the difficulty recruiting researchers willing to develop C$++$ software. The decision was made to reimplement SmartLiver using the SciKit-Surgery libraries.

One of the design considerations of NifTK [3] was to keep things modular, so in theory code can be separated out when trying to develop a clinical application. Rather than considering NifTK as a single dependency (Fig. 1), we can look at the second-order dependencies of the SmartLiver plugin in terms of the individual modules of NifTK. Figure 2 shows a comparison between the first- and second-order dependencies of SmartLiver implemented using SciKit-Surgery and the modular dependencies when using NifTK. In terms of the number and type of dependencies, they are similar. Most of size of both applications comes from QT, VTK, and OpenCV. However, the application built using NifTK has significantly more cross-dependence amongst its direct dependencies.

Figure 2 highlights cross-dependencies (dependencies between first-order dependencies) using dashed lines. Cross-dependencies are problematic as they reduce the orthogonality of the code [14]. Making a change to one of these libraries is likely to lead to unintended consequences in a dependent library. The SciKit-Surgery-based application actually has more(9) cross-dependencies than the NifTK implementation(6). With one exception (surgeryvtk to surgeryimage) however, the SciKit-Surgery cross-dependencies are to third-party libraries that are unlikely to require changes during development. Whilst it is not impossible that a significant change to one of these libraries could break the application, such a change could at least be managed by locking the library version. In contrast, it is very likely that changes will be required within the direct dependencies that form part of either NifTK or SciKit-Surgery. The NifTK implementation has four cross-dependencies between these libraries compared to one for the SciKit-Surgery implementation. Looking at the NifTK dependency diagram, we can see that changes within either IGIDataSources or IGIGui could have unintended effects on either their direct or second-order dependents. This makes development and maintenance significantly more difficult.

**Fig. 3:**
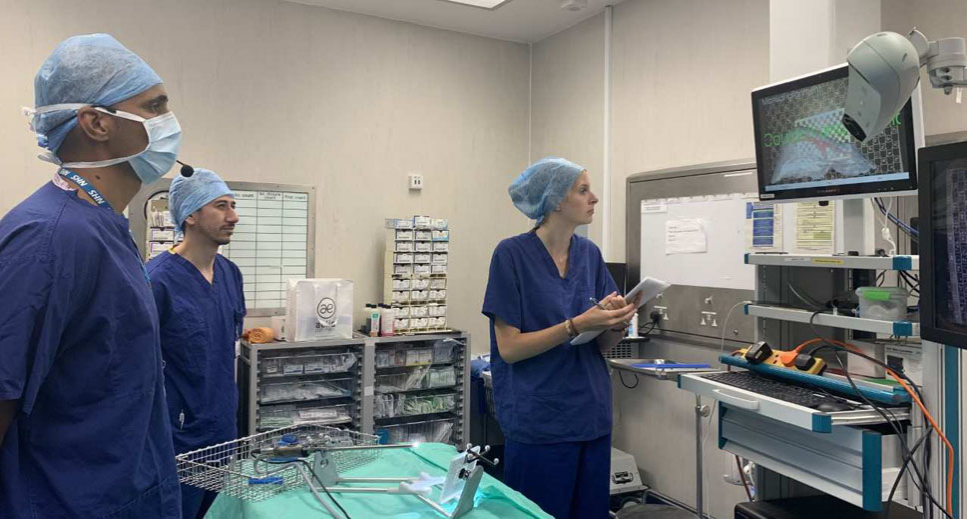
The SmartLiver system being tested in theatre with a voice-controlled user interface. The SciKit-Surgery architecture allows the SmartLiver system to be easily connected to the scikit-surgeryspeech library, to test the effectiveness of voice control for calibration and manual alignment

Figure 3 shows the current SmartLiver system being tested in theatre with a novel voice-based user interface. The modular architecture of the SciKit-Surgery libraries made it straightforward to connect the scikit-surgeryspeech library to the SmartLiver application.

The SmartLiver application has now entered WEISS’s ISO-13485 Quality Management System, to be developed into an approved clinical application. The SciKit-Surgery libraries used by SmartLiver will remain outside the quality management system, treated as software of unknown provenance (SOUP). SciKit-Surgery’s smaller size and more linear dependency tree make it significantly easier to maintain it and the SmartLiver application, supporting goal 2 of the SciKit-Surgery libraries.

### SnappySonic

**Fig. 4:**
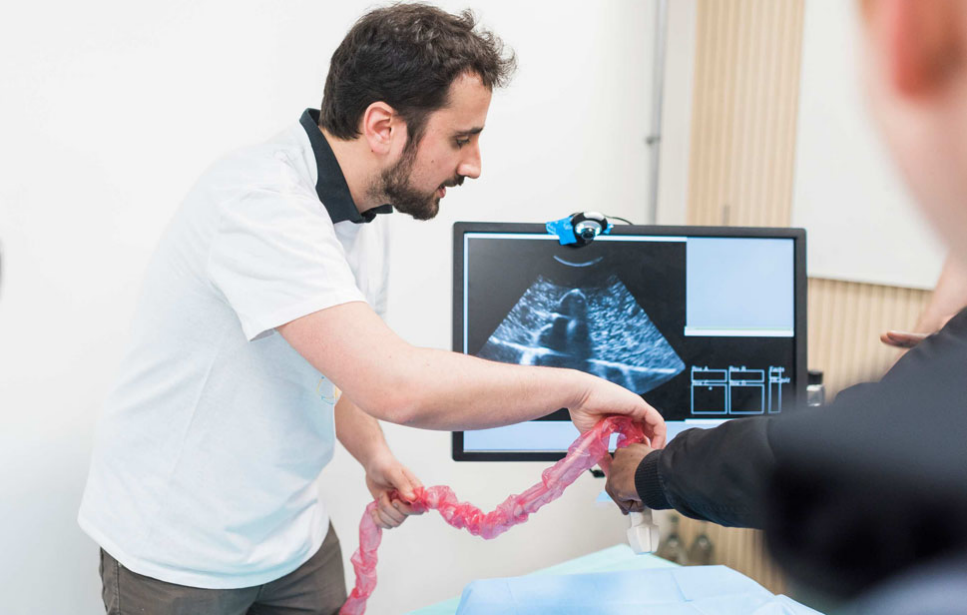
SnappySonic in use at the Science of Surgery public engagement event. An obsolete ultrasound probe is tracked using OpenCV’s ArUco marker libraries. The position of the probe is used to show an image from a pre-recorded ultrasound buffer, giving the appearance of live acquisition. Image by James Tye

The SnappySonic ultrasound simulator[21] is used here to demonstrate the ability of the SciKit-Surgery libraries to support goals 1 and 3 from Tables 2 and 3. The SnappySonic software was developed to support a “serious game” [11] style ultrasound demonstration at public engagement events. Development of the SnappySonic application highlights some of the benefits of SciKit-Surgery. The application itself went from idea to basic demo within two working days, between the 27th of March 2019 and the 2nd of April 2019, utilising the Python Template, scikit-surgeryutils, and scikit-surgeryarucotracker. The complete demonstration (including hardware) was tested internally on the 7th of April and deployed at the “Science of Surgery” event on the 12th of April 2019. This gave a good demonstration of the SciKit-Surgery libraries’ ability to support goal 1 from Tables 2 and 3. The application consists of 1675 lines of code. Figure 4 shows the application in use.

After deployment at the Science of Surgery event, it was decided that the software was of potentially broader use and should be maintained and published on PyPI. The focus now changed to ensuring adequate documentation and test coverage. This process took around a week of work, fitted around other projects, and on the 28th of May the project was made available on PyPI. In its entirety, the application went from idea to a sustainable open source application in around 2 months, illustrating the SciKit-Surgery libraries’ ability to support goal 3 from Tables 2 and 3.

## Discussion

The SciKit-Surgery libraries form a collection of tools for surgical navigation that can be bound together with Python, making use of standard graphical user interface components to rapidly develop novel applications.

In Sect. 5, we compared the occurrence of cross-dependencies in two implementations of the same application (SmartLiver). The implementation using a single modular platform (NifTK) exhibited substantially more cross-dependencies. These make development more difficult and discourage new developers from contributing as it is harder to predict the likely effects of any changes. It could be argued that such cross-dependencies should be avoided by more robust software engineering process within the development team; however, this goes against a large part of what we are trying to achieve with SciKit-Surgery.

We do not wish to provide a set of libraries developed by a small team where contributions are closely monitored and checked for quality, but rather a set of libraries with a large base of contributors that avoid cross-dependencies by design. Whilst NifTK is modular, integration testing is only performed on the full platform; thus, cross-dependencies are not immediately obvious, and it is tempting for developers to add dependencies between modules to avoid reimplementing similar functions. By testing and deploying every SciKit-Surgery library individually, the introduction of cross-dependencies should be reduced

It will be interesting ongoing work to investigate the occurrence of cross-dependencies and duplication within the SciKit-Surgery libraries and compare this to similar features in more monolithic platforms. It is our hope to decentralise the development and maintenance of much of SciKit-Surgery. Decentralisation will enable ongoing growth, but will come at the cost of control and possible divergence within the libraries. Defining key data types and interfaces within scikit-surgerycore may minimise this.

Development is ongoing with a growing base of contributors and users. Current work is developing graphics processing unit accelerated libraries that use the same NumPy interfaces, so that surgical video can be processed in real time. However, by keeping libraries independent as far as practicable, we are able to rapidly change our development plans in response to the needs of our user base.

Investigation into the best way to deploy clinical applications built using Python is ongoing. Whilst Python packages provide a flexible tool for researchers, distribution to clinical users needs to be simpler. For most interventional applications, this is not an issue, as the software would be delivered pre-installed on a dedicated clinical-grade computer. Stand-alone installers for specific applications could also be created using tools like pyinstaller.^6^

As part of our analysis, we found that typical surgical applications and their modular dependencies run from 1000 to 3000 lines of code. We speculate that this is the amount of code a typical researcher can write and maintain to support their research objectives.

## Conclusions

We have presented the SciKit-Surgery libraries, a set of largely stand-alone libraries to support research innovation and translation in surgical navigation. The SciKit-Surgery libraries have been under active development for approximately 1 year. In that time, we have demonstrated their suitability for use in surgical navigation and related applications. We have had significantly more success with engaging staff and students in library development than with other platforms (NifTK), which we attribute to the simplified structure and use of Python rather than C$++$. At present, the SciKit-Surgery libraries do not support the same amount of functionality as existing medical imaging and IGI platforms; however, we have shown how SciKit-Surgery can be rapidly developed to support new functionality.

Interested readers are directed to the first two SciKit-Surgery tutorials;

- The SciKit-Surgery augmented reality tutorial https://snappytutorial01.readthedocs.io,
- The SciKit-Surgery library development tutorial https://snappytutorial02.readthedocs.io,
- And the SciKit-Surgery wiki https://github.com/UCL/scikitsurgery/wiki.

## Footnotes

1 https://github.com/AlDanial/cloc.

2 www.pypi.org.

3 www.pylint.org.

4 https://pypi.org/project/yunguanfu-mil3id2019/.

5 https://www.visiblepatient.com.

6 https://www.pyinstaller.org/.
